# Label-Free Homogeneous Electrochemical Aptasensor Based on Size Exclusion/Charge-Selective Permeability of Nanochannel Arrays and 2D Nanorecognitive Probe for Sensitive Detection of Alpha-Fetoprotein

**DOI:** 10.3390/molecules28196935

**Published:** 2023-10-05

**Authors:** Yue Zhang, Shiyue Zhang, Jiyang Liu, Dongyuan Qin

**Affiliations:** 1 Department of Hepatology, Taiyuan Third People’s Hospital, Taiyuan 030012, China; 2 School of Chemistry and Chemical Engineering, Zhejiang Sci-Tech University, Hangzhou 310018, China; 3 Shanxi Bethune Hospital, Shanxi Academy of Medical Sciences, Tongji Shanxi Hospital, Third Hospital of Shanxi Medical University, Taiyuan 030032, China

**Keywords:** homogeneous aptamer sensor, electrochemical detection, labeling-free and immobilization-free, nanochannel array-modified electrode, two-dimensional nanorecognition probe

## Abstract

The labeling-free and immobilization-free homogeneous aptamer sensor offers advantages including simple operation, low cost, and high sensitivity, demonstrating great potential in rapid detection of tumor biomarkers in biological samples. In this work, a labeling-free and immobilization-free homogeneous aptamer sensor was conveniently fabricated by combining size exclusion and charge-selective penetration of a nanochannel-modified electrode and two-dimensional (2D) nanorecognition probe which can realize selective and highly sensitive detection of alpha-fetoprotein (AFP) in serum. Vertically ordered mesoporous silica film (VMSF) with ultra-small, uniform, and vertically aligned nanochannels was easily grown on the simple, low-cost, and disposable indium tin oxide (ITO) electrode. Through π-π interaction and electrostatic force, the AFP aptamer (Apt) and electrochemical probe, tris(bipyridine)ruthenium(II) (Ru(bpy)_3_^2+^), were coloaded onto graphene oxide (GO) through simple incubation, forming a 2D nanoscale recognition probe (Ru(bpy)_3_^2+^/Apt@GO). Owing to the size exclusion effect of VMSF towards the 2D nanoscale probe, the electrochemical signal of Ru(bpy)_3_^2+^/Apt@GO could not be detected. In the presence of AFP, the specific binding of AFP to the aptamer causes the dissociation of the aptamer and Ru(bpy)_3_^2+^ from GO, resulting in their presence in the solution. The efficient electrostatic enrichment towards Ru(bpy)_3_^2+^ by negatively charged VMSF allows for high electrochemical signals of free Ru(bpy)_3_^2+^ in the solution. Linear determination of AFP ranged from 1 pg/mL to 1000 ng/mL and could be obtained with a low limit of detection (LOD, 0.8 pg/mL). The high specificity of the adapter endowed the constructed sensor with high selectivity. The fabricated probe can be applied in direct determination of AFP in serum.

## 1. Introduction

Liver cancer seriously threatens human health worldwide because of its high mortality rate [1]. Early diagnosis is crucial for the prevention and treatment of liver cancer [2]. On one hand, early diagnosis can increase the opportunity for patients to receive timely and effective treatments such as surgical resection, radiofrequency ablation, and transarterial chemoembolization, thereby improving the cure rate. On the other hand, early treatment can effectively control tumor growth and metastasis, reducing the risk of recurrence and metastasis. Therefore, early diagnosis and treatment of liver cancer are essential for improving prognosis and survival rates. Tumor biomarkers refer to molecules or substances associated with tumors detected in human tissues, blood, urine, or other biological samples [3,4]. Currently, alpha-fetoprotein (AFP) is one of the most common and important tumor biomarkers for liver cancer [5,6]. AFP is a protein produced by the liver and fetus, primarily synthesized by the placenta during embryonic development and entering the fetal bloodstream, playing a supportive role in normal fetal development. In normal adults, the level of AFP is usually very low. The level of AFP in serum can be used to screen high-risk populations (e.g., those with a family history or occupational exposure to carcinogens), assist in early detection and diagnosis of liver cancer, monitor treatment efficacy, and predict prognosis. Various methods have been developed to detect AFP, the most commonly used methods among which are enzyme-linked immunosorbent assay (ELISA), chemiluminescent immunoassay, or magnetic bead separation combined with electrochemiluminescence (ECL) detection [7,8]. However, these methods often suffer from intensive experimental procedures, long analysis time, relatively expensive reagents, and antibody deactivation during transportation and storage. Nucleic acid aptamers with antibody-like functions have advantages such as easy synthesis/modification, low cost, and high stability and can be reused and stored for long periods [9]. Developing efficient aptamer sensors for rapid, convenient, and highly sensitive detection of AFP in serum holds significant importance.

Electrochemical detection can obtain accurate analytical results by measuring the changes in electrochemical signals such as current, voltage, or charge [10,11]. With their high sensitivity and rapid response, electrochemical sensors have great potential in AFP detection [12]. However, traditional aptamer-based electrochemical sensors often use solid-phase sensing interfaces, which require immobilization of recognitive probes or signal probes on the surface of the underlying electrode. This immobilization process is frequently complex, and the low probe density and uncontrolled orientation result in low probe activity and poor recognition efficiency and reproducibility. In contrast, labeling-free and immobilization-free homogeneous aptamer sensors are a detection method that does not require the immobilization of recognition probes on the electrode surface or any modification of the aptamer [13,14]. Compared with the methods using labeled or immobilized probes, the labeling-free and immobilization-free strategy has lower cost, shorter and simpler steps, and greatly reduces experimental difficulty. In addition, the recognition of analytes occurs in a homogeneous solution, with less steric hindrance, resulting in relatively high binding efficiency and recognition rate between the target and recognition probe. Therefore, homogeneous aptamer electrochemical sensors display great potential owing to simple detection process, very low cost, high sensitivity, as well as ease of development into portable test kits, demonstrating great potential in rapid AFP detection. However, when homogeneous electrochemical aptamer sensors are used for AFP analysis in serum, they still face challenges such as complex matrix and low concentration of AFP in early-stage liver cancer. For example, proteins and other biomacromolecules or large particles in serum are prone to nonspecific adsorption on the electrode surface, leading to electrode fouling and low sensitivity, accuracy, and reproducibility of the detection. Additionally, in the early stages of liver cancer, the concentration of AFP is often low, so signal amplification strategies are commonly employed to enhance detection sensitivity. Therefore, direct and highly sensitive electrochemical detection of AFP in serum by achieving efficient signal amplification and improving the resistance to complex matrix is highly desirable.

Nanomaterials have large specific surface areas and unique electrochemical/optical/magnetic properties that can enhance the sensitivity and selectivity of sensors [15,16,17,18,19]. The introduction of nanomaterials to functionalize the electrode surface is an attractive strategy to enhance the detection performance of electrochemical sensors [20,21,22]. Among these materials, porous materials have received considerable attentions because they have high specific surface area, diverse structure, and adjustable pore size [23,24,25]. In recent years, mesoporous nanomaterials have shown great potential in catalysis, energy storage, sensing, and adsorption or separation [26,27]. Vertically ordered mesoporous silica film (VMSF) is attractive due to its ultrathin nanoscale thickness and unique mesoporous structure [28]. VMSF consists of array with ultra-small, uniform, and vertically aligned nanochannels, providing the advantages of adjustable nanochannel diameter (1.3~11.8 nm), thickness (50~200 nm), high porosity (10^12^ nanochannels/μm^2^), and large specific surface area [29,30,31]. Unlike the mass transfer in micrometer-scale channels, nanochannels possess molecular-level electrostatic and size-sieving capabilities. They can introduce selective sieving capabilities at the molecular level for the substrate electrode, allowing only small molecules with charge-matching and size-matching properties to pass through, significantly reducing the interference of coexisting electroactive substances. On the one hand, the silica material of VMSF is electrically insulating, and the nanochannels with ultra-small diameter have size-sieving capability, allowing only small molecules to pass through while effectively blocking larger biomolecules (cells, bacteria, proteins, DNA, polysaccharides, etc.) and solid particles, eliminating fouling from proteins and solid particles on the underlying electrode [32,33,34,35]. At the same time, the ionization of silanol groups (Si-OH) in the silica framework of VMSF (p*K*_a_~2) results in a negatively charged surface of the nanochannels, enabling the electrostatic enrichment of positively charged small molecules [36,37,38,39]. Combined with the high specific surface area of the nanochannel array, significant enrichment of positively charged electrochemical probes can be achieved. Therefore, VMSF-modified electrodes can effectively achieve the separation or enrichment of substances in complex matrices through size exclusion and charge-selective penetration, enabling integrated separation, enrichment, and detection and demonstrating great potential in analysis of tumor biomarkers [40,41,42].

In this work, a labeling-free and immobilization-free homogeneous electrochemical aptamer sensor was fabricated for direct and highly sensitive detection of AFP in serum by combining nanoscale recognition probes with VMSF, which possesses size exclusion and charge-selective penetrability. Through π-π interaction and electrostatic force, the AFP aptamer (Apt) and electrochemical probe, tris(bipyridine)ruthenium(II) (Ru(bpy)_3_^2+^), were coloaded onto graphene oxide (GO), forming a two-dimensional (2D) nanoscale recognition probe (Ru(bpy)_3_^2+^/Apt@GO). VMSF was conveniently grown when an indium tin oxide (ITO) electrode was employed as the supporting electrode. Owing to the size exclusion effect of VMSF towards the prepared 2D nanoscale probe, the electrochemical signal of Ru(bpy)_3_^2+^/Apt@GO could not be detected. For the detection of AFP, the specific binding of AFP to the aptamer causes the dissociation of the aptamer and Ru(bpy)_3_^2+^ from GO, resulting in their presence in the solution. The efficient electrostatic enrichment towards Ru(bpy)_3_^2+^ by VMSF results in high electrochemical signals of free Ru(bpy)_3_^2+^, leading to sensitive electrochemical detection of AFP. As the homogeneous detection system eliminates the need for the immobilization or labeling of recognition and signal probes, the fabricated sensor demonstrates advantages including a simple construction process, easy detection process, low cost, and high detection performance, showing great potential in rapid, sensitive determination of tumor biomarkers with electrochemical sensors.

## 2. Results and Discussion

### 2.1. Strategy for the Construction of Labeling-Free and Immobilization-Free Homogeneous Electrochemical Aptasensor

**Figure 1:**
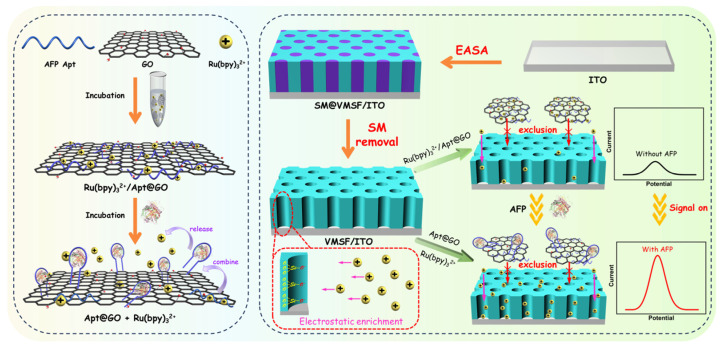
Illustration of the fabrication of labeling-free and immobilization-free homogeneous electrochemical aptasensor for detecting AFP by integrating nanoscale recognition probes with nanochannel array-modified electrode. The left panel shows the preparation of nanoscale recognition probe and its binding to AFP. The right panel illustrates the fabrication of VMSF/ITO electrode and the process of AFP detection.

Figure 1 illustrates the construction of a labeling-free and immobilization-free homogeneous electrochemical aptasensor for detecting AFP by integrating nanoscale recognition probes with nanochannel array-modified electrodes. In this approach, a GO, AFP aptamer, and electrochemical probe, Ru(bpy)_3_^2+^, are incubated together. Through π-π interactions and electrostatic forces, the AFP aptamer and Ru(bpy)_3_^2+^ are coloaded onto GO, forming a 2D nanoscale recognition probe, Ru(bpy)_3_^2+^/Apt@GO. An electrochemical-assisted self-assembly method is used to directly grow VMSF on a simple, inexpensive, and disposable ITO electrode, which serves as the working electrode for subsequent detection. The growth mechanism of VMSF involves the use of cationic surfactant (cetyltrimethylammonium bromide, CTAB) micelle (SM) as a soft template for the hydrolysis and condensation reactions of siloxane. Electrochemical anodization with a negative potential applied to the electrode enables rapid growth of VMSF within 5–30 s, forming nanochannels vertically oriented to the electrode surface and filled with CTAB micelles. After removing the micelles in hydrochloric acid solution (0.1 M in ethanol), an electrode with an open nanochannel is obtained.

VMSF possesses ultra-small nanochannel structures that can hinder the passage of large-sized substances or macromolecules. This is because the diameter of a VMSF nanochannel is close to the Debye length, enabling size-sieving capability on a molecular level. Therefore, VMSF can block large substances, particulate matter, and 2D nanomaterials in complex solutions. Consequently, when a 2D nanomaterial probe is used as the matrix for homogeneous analysis, large-sized substances or macromolecules can be impeded, allowing only small molecule probes to penetrate the channels. In the solution containing Ru(bpy)_3_^2+^/Apt@GO, only a small amount of unbound Ru(bpy)_3_^2+^ is free in the solution, resulting in low electrochemical signals. When the target molecule AFP is present, the aptamer binds to the target, causing it to detach from the GO surface. Simultaneously, because of the significant electrostatic attraction between Ru(bpy)_3_^2+^ and the aptamer, Ru(bpy)_3_^2+^ can also dissociate from GO and remains free in the solution. Since VMSF exhibits strong electrostatic enrichment of Ru(bpy)_3_^2+^ and size exclusion towards GO-supported probes, the Ru(bpy)_3_^2+^ in the solution can be adsorbed, enhancing the electrochemical signal. By monitoring the changes in the electrochemical signal on VMSF-modified electrode, sensitive determination of AFP can be performed.

### 2.2. Surface Morphology and Charge Selectivity of VMSF-Based Electrode

**Figure 2:**
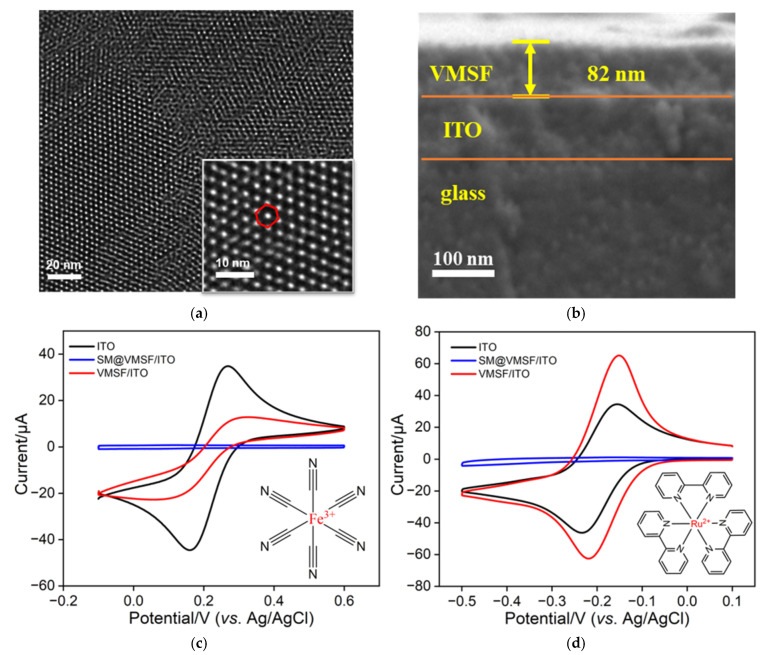
(**a**) TEM image of surface of VMSF. Inset is the corresponding high-resolution TEM image. The circled portion represents a hexagonal structure formed by the arrangement of the nanochannels. (**b**) Cross-sectional SEM of VMSF/ITO electrode. (**c**,**d**) CV curves measured using different electrodes in KHP (50 mM, pH = 4) + Fe(CN)_6_^3−^ (0.5 mM, (**c**)) or Ru(NH_3_)_6_^3+^ (0.5 mM, (**d**)). Scan rate is 100 mV/s.

The surface morphology and film thickness of VMSF were characterized using TEM and SEM. Figure 2a shows a top-view TEM image of VMSF. It can be observed that the VMSF has no apparent defects or cracks, and the pores (nanochannels) are evenly arranged. The inset image is a magnified TEM image, revealing a hexagonal structure of the nanochannels. Measurement with ImageJ software indicated a pore diameter of 2.5 nm and a pore density as high as 8.4 × 10^12^ pores/cm^2^, corresponding to a porosity of 41%. Figure 2b presents the SEM image of the cross-sectional part of the VMSF-functionalized ITO electrode. It demonstrates a clear and three-layered interface including the top VMSF, the middle ITO, and the bottom glass layer, confirming the successful growth of VMSF on an ITO electrode. The thickness of the VMSF film is approximately 82 nm.

To investigate the integrity and charge-based permeability selectivity of VMSF, two electrochemical probes, including Fe(CN)_6_^3−^ and Ru(NH_3_)_6_^3+^ with opposite charges, were selected to investigate their cyclic voltammetry (CV) characteristics on different electrodes. As shown in Figure 2c,d, a bare ITO electrode and VMSF-modified electrode before and after the removal of surfactant micelles were investigated. As a commonly used conductive electrode, electrochemical probes exhibit distinct oxidation-reduction peaks on the ITO electrode. In the presence of a surfactant micelle template, the SM@VMSF/ITO electrode exhibits no Faradaic currents for Fe(CN)_6_^3−^ as well as Ru(NH_3_)_6_^3+^ probes. This indicates that the presence of SM inside the nanochannels prevents the probe molecules from reaching the ITO surface and undergoing electrochemical reactions. In addition, the result also proves that the formed SM@VMSF/ITO is intact without cracks or defects. The VMSF/ITO electrode exhibits oxidation-reduction signals for two different electrochemical probes. This can be attributed to the presence of open, high-density nanochannels after the removal of micelles, ensuring the diffusion of small molecular probes to the electrode surface. In addition, the negatively charged Fe(CN)_6_^3−^ shows a lower (31%) electrochemical signal compared to that on bare ITO electrode, indicating that the nanochannels repulse the Fe(CN)_6_^3−^ probe. On the other hand, the positively charged Ru(NH_3_)_6_^3+^ probe exhibits a higher (176%) peak current than that obtained on bare electrode. Thus, a negatively charged probe exhibits a lower signal while the positively charged probe has a higher signal compared with that on the supporting electrode. This demonstrates the charge-based selective permeability of VMSF nanochannels. This might be ascribed to the negatively charged surface of VMSF. Briefly, due to the presence of silanol groups (p*K*_a_ ≈ 2–3) on the surface of nanochannels, which are prone to protonation, the VMSF surface becomes negatively charged, leading to electrostatic repulsion towards negatively charged electrochemical probes while electrostatically enriching positively charged probes.

### 2.3. Synthesis and Characterization of 2D Nanoscale Recognition Probe

**Figure 3:**
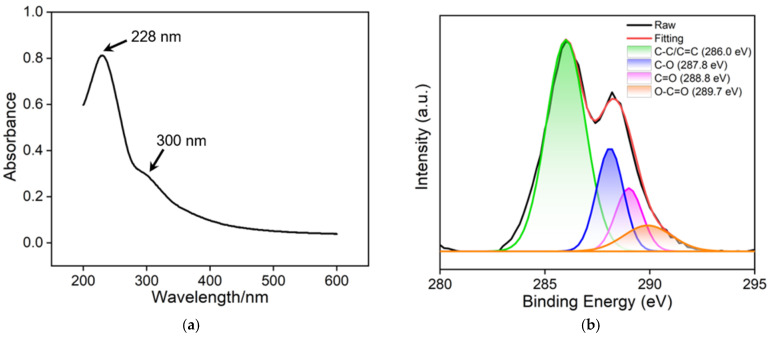

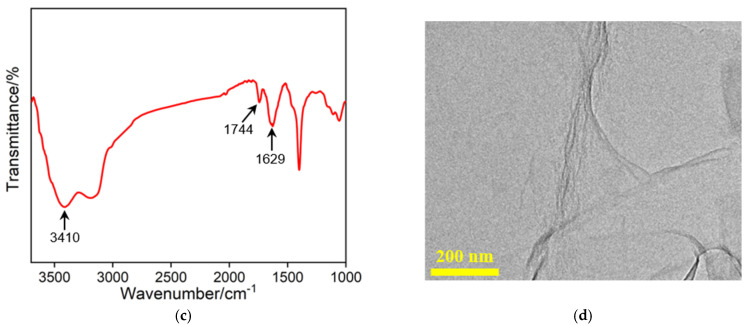
(**a**) UV-Vis absorption spectrum of GO. (**b**) High-resolution C1s spectrum of GO. (**c**) FTIR spectrum of GO. (**d**) TEM image of GO.

Nanocarbon materials, such as graphene or its oxide, carbon nanotubes, carbon dots/graphene quantum dots, and nanodiamonds, are the most widely used functional nanomaterials. Nanocarbon materials have a large specific surface area which provides more reaction interfaces, thereby facilitating catalysis, adsorption, sensing, and other reaction processes. Easy preparation, controllable chemical functional groups, and excellent water dispersibility make graphene oxide the most widely used 2D nanomaterial. In this study, GO was used as a carrier for both recognition aptamer ligands and electrochemical signal probes. From the UV-Vis absorption spectrum in Figure 3a, it can be observed that GO exhibits two absorption peaks, at 226 nm and 300 nm, resulting from the π-π* transition absorption band of unsaturated carbon bonds and the n-π* transition absorption band in the conjugated structure, respectively. XPS was used to investigate the surface functional groups of GO. Figure 3b shows the high-resolution C1s spectrum, which displays four characteristic functional groups including C-C/C=C (sp^2^ carbon) at 286.0 eV, C-O at 287.8 eV, C=O at 288.8 eV, and O-C=O at 289.7 eV, respectively. Furthermore, the functional groups of GO were characterized by FTIR spectroscopy, as shown in Figure 3c. The absorption peaks at 1744 cm^−1^ and 1629 cm^−1^ correspond to the stretching vibrations of C=O and C=C, respectively, while the absorption peak at 3419 cm^−1^ is attributed to the stretching vibration of O-H. These results confirm the presence of a large number of oxygen-containing functional groups in GO, which facilitates its dispersion in water. The morphology of GO was characterized by TEM, as depicted in Figure 3d. It can be observed that GO has a single-layered structure with folding. The single-layered structure enhances the specific surface area, which is beneficial for the loading of functional molecules.

**Figure 4:**
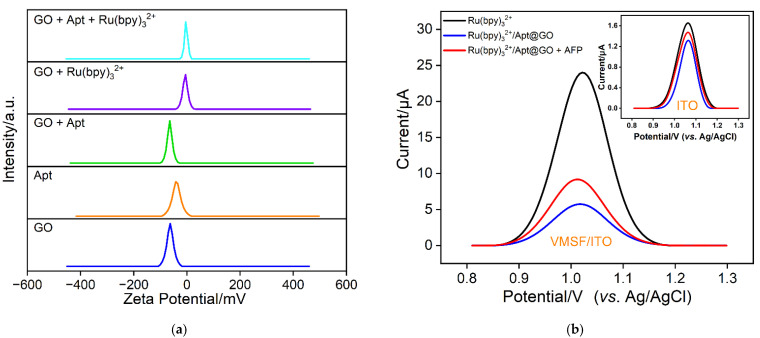
(**a**) The zeta potential of nanomaterials in different incubation solutions. (**b**) DPV curves obtained on ITO or VMSF/ITO electrode in Ru(bpy)_3_^2+^, Ru(bpy)_3_^2+^/Apt@GO nanoprobe in absence or presence of AFP.

To study the preparation process of the Ru(bpy)_3_^2+^/Apt@GO nanocomposite probe, the charge characteristics of nanomaterials in different incubation solutions were investigated using Zeta potential. From Figure 4a, it can be observed that the Zeta potentials of the GO and AFP adapter are approximately −62.9 mV and −39.4 mV, indicating strong negative charge. The strong negative charge of GO is attributed to the presence of abundant oxygen-containing groups, for instance -COOH, -OH, etc. These groups deprotonate and result in a negatively charged surface of GO. The AFP adapter, due to the presence of a phosphoric acid molecular backbone, also exhibited strong negative charge. When GO is incubated with Apt, the Zeta potential becomes −64.5 mV, indicating an even stronger negative charge compared to pure GO. This suggests the binding of AFP adapter molecules to GO, possibly attributed to the π-π interaction between the nitrogenous bases of aptamer and the sp^2^ carbon framework of GO. When GO is co-incubated with Ru(bpy)_3_^2+^, the Zeta potential of the nanomaterial shifts significantly towards positive values. This can be attributed to the electrostatic adsorption between Ru(NH_3_)_6_^3+^ and GO nanosheets, which allows Ru(NH_3_)_6_^3+^ to bind to GO and significantly reduce the negative charge of the resulting nanocomposite. When GO is co-incubated with Apt and Ru(NH_3_)_6_^3+^, the negative charge of Ru(NH_3_)_6_^3+^/Apt@GO is slightly lower than that of Ru(NH_3_)_6_^3+^@GO, owing to the negative charge of Apt. The above results demonstrate the convenient synthesis of the Ru(NH_3_)_6_^3+^/Apt@GO nanocomposite probe.

### 2.4. Feasibility of AFP Detection Using Homogeneous Aptamer Sensor

The homogeneous aptamer sensor can be fabricated based on nanoprobes and a nanochannel-modified electrode, which can achieve electrochemical detection of AFP by monitoring the electrochemical signal changes of Ru(bpy)_3_^2+^ before and after AFP binding. As shown in Figure 4b, Ru(bpy)_3_^2+^ exhibits a significant signal on the VMSF/ITO electrode. As displayed, the peak current on the VMSF/ITO electrode is nearly 10 times higher than that obtained on the corresponding ITO electrode. This is attributed to the negatively charged structure of VMSF nanochannels, which allows for efficient electrostatic enrichment of Ru(bpy)_3_^2+^, leading to high electrochemical signals. However, when Ru(bpy)_3_^2+^ is incubated with GO and Apt to form the Ru(bpy)_3_^2+^/Apt@GO nanocomposite probe, the concentration of free Ru(bpy)_3_^2+^ in solution decreases due to its binding to GO. Furthermore, the ultra-small VMSF nanochannels exhibit a size exclusion effect on the 2D Ru(bpy)_3_^2+^/Apt@GO nanoprobes, preventing Ru(bpy)_3_^2+^ on the nanoprobe from making direct contact with the underlying electrode surface and generating an electrochemical signal. As a result, the VMSF/ITO electrode can only detect free Ru(bpy)_3_^2+^ in the solution, leading to a decrease in peak current. On the other hand, changes in the electrochemical response of Ru(bpy)_3_^2+^ on the ITO electrode before and after the formation of the nanoprobe are not significant. When AFP is added to the solution, the nanoprobes specifically recognize AFP through aptamer–target binding. The binding of AFP causes the aptamer to detach from GO, altering the charge properties of the nanocomposite and resulting in the release of some Ru(bpy)_3_^2+^ into the solution. Consequently, the concentration of free Ru(bpy)_3_^2+^ in the solution changes. The signal change of Ru(bpy)_3_^2+^ on VMSF/ITO is also remarkably higher than that of the supporting ITO. Clearly, the nanochannel-modified electrode can sensitively detect the signal changes of Ru(bpy)_3_^2+^ owing to its strong electrostatic enrichment effect. These results confirm the feasibility of a homogeneous aptamer sensor based on a nanochannel-modified electrode and 2D recognition probes for AFP detection.

### 2.5. Electrochemical Determination of AFP Using the Fabricated Homogeneous Aptamer Sensor

**Figure 5:**
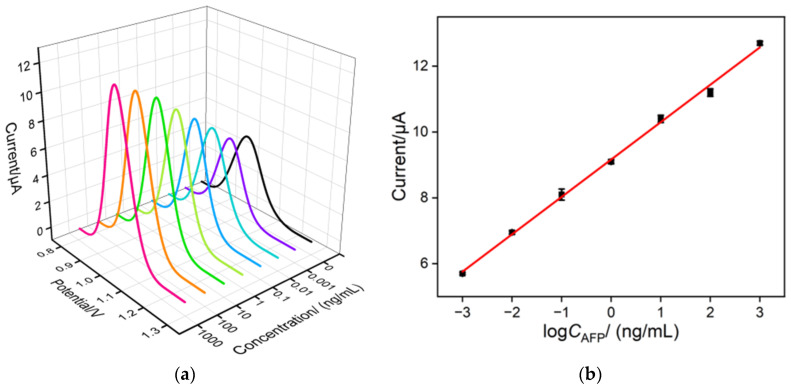
(**a**) DPV curves for detecting different concentrations of AFP using the constructed homogeneous aptamer sensor. (**b**) The corresponding linear calibration curve.

Figure 5a shows the DPV curves for detecting different concentrations of AFP using the constructed homogeneous aptamer sensor. As shown, the anodic peak current of Ru(bpy)_3_^2+^ increases with increasing AFP concentration. This is because, after the addition of AFP, the specific recognition between the aptamer and AFP causes the release of the adsorbed Ru(bpy)_3_^2+^ from the Ru(bpy)_3_^2+^/Apt@GO complex into the solution. Figure 5b demonstrates that within the range of 1 pg/mL to 1000 ng/mL AFP concentration, there is a good linear relationship between the anodic peak current (*I*_EC_) and the logarithm of AFP concentration (log*C*_AFP_) (*I*_EC_ = 1.14 log*C*_AFP_ + 9.16, R^2^ = 0.995). The detection limit (LOD) calculated based on a signal-to-noise ratio of three (S/N = 3) is 0.8 pg/mL. Table 1 summarizes the comparison between AFP detection performance using different electrodes through photoelectrochemical (PEC), electrochemical (EC), or electrochemiluminescence (ECL) detection [43,44,45,46,47,48]. The LOD of the fabricated sensor is lower than that obtained using the AFP antibody/conjugated polymers/titanium dioxide/fluorine-doped tin oxide conductive glass electrode (Ab/CP/TiO_2_/FTO) [43], sandwich analysis based on poly-(2-hydroxyethyl methacrylate)-g-graphene/the second antibody/AFP/BSA/the first antibody/gold nanoparticle-reduced graphene oxide-modified GCE (P(VT-co-HEMA)-g-GO/Ab_2_/AFP/BSA/Ab_1_/AuNPs-rGO/GCE) [44], metal–organic framework-antibody/AFP/BSA/aptamer-SH/gold nanoparticle/flexible polydimethylsiloxane-modified GCE (MOF-Ab/AFP/BSA/Apt-SH/Au/PDMS/GCE) [45], or antibody/carbodiimide hydrochloride-N-hydroxysuccinimide/ferroferric oxide/carboxylated carbon nanotubes/gold nanoparticle-modified GCE (Ab/EDC-NHS/Fe_3_O_4_/MWCNTs-COOH/AuNPs/GCE) [46] but higher than that obtained on AFP/BSA/AFP antibody/reduced graphene oxide@gold nanoparticle@Ru(bpy)_3_^2+^-doped silica nanoparticle-modified GCE (AFP/BSA/Ab/rGO@Au@Ru–SiO2/GCE) [47] and fluorescent microspheres based on 4-vinylphenylboronic acid and divinylbenzene-AFP/AFP antibody/Ti, N/Mg co-doped carbon dot-modified GCE (Poly(DVB-Co-PBA)-AFP/Ab/Ti,Mg@N-CDs/GCE) [48]. Compared with other electrodes that need antibody/DNA to be fixed at the recognition interface or sandwich detection modes, the homogeneous electrochemical sensor constructed by this method has the advantages of simple preparation and high sensitivity.

### 2.6. Selectivity, Reproducibility, and Reuse of the Aptamer Sensor and Real Sample Analysis

**Figure 6:**
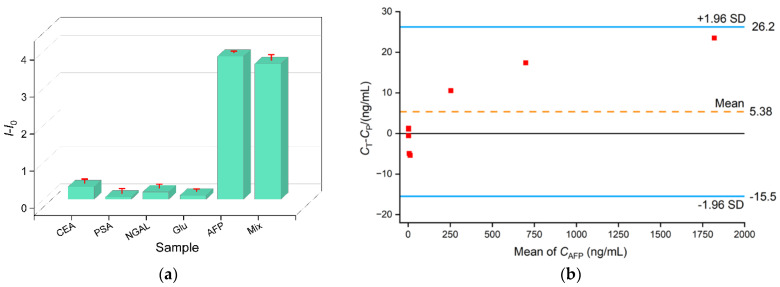
(**a**) The DPV signal difference (*I*-*I*_0_) before (*I*_0_) and after (*I*) Ru(bpy)_3_^2+^/Apt@GO incubates with CEA (0.1 ng/mL), PSA (0.1 ng/mL), NGAL (0.1 ng/mL), Glu (10 μM), AFP (0.1 ng/mL), or their mixture. (**b**) The Bland–Altman scatter plot in Bland–Altman analysis of the detection results obtained using the fabricated homogenous aptamer sensor and Roche ECL analyzer.

The selectivity of the constructed aptamer sensor was examined. Other tumor markers including CEA, PSA, or NGAL were separately incubated with the Ru(bpy)_3_^2+^/Apt@GO nanoprobe. It is acknowledged that serum contains high concentrations of glucose. Thus, the effect of glucose on detection was also investigated. As shown in Figure 6a, the addition of one of the above substances does not cause remarkable change in the electrochemical signal of Ru(bpy)_3_^2+^. Only AFP or the mixture of AFP with the above substances results in a significant increase in the electrochemical signal of Ru(bpy)_3_^2+^, confirming the selectivity of the nanoprobe and the antifouling performance of the VMSF/ITO electrode. To assess the reproducibility of the fabricated sensor, five electrodes were separately prepared, using the same method, and employed for the detection of AFP (10 ng/mL); the relative standard deviation (RSD) of the obtained results was 2.9%, demonstrating good reproducibility. The constructed sensor can be easily regenerated. After use, the electrode can be immersed in a 50% ethanol–water solution for 5 min to complete the electrode regeneration. The regenerated electrode can continue to be used for AFP detection. When the electrode was reused five times for AFP detection (10 ng/mL), the RSD of the results was 3.7%, demonstrating that VMSF remains stable, intact, and does not crack during use.

The homogenous aptamer sensor was utilized to measure the AFP levels in the serum of healthy individuals and liver cancer patients. A total of seven samples were tested, and the detection values (*C*t) were compared with the results (*C*p) obtained from a Roche electrochemical luminescence analyzer using the Bland–Altman method. Figure 6b shows the corresponding Bland–Altman scatter plot, where the *y*-axis represents the difference between the two detection results (*C*t-*C*p), and the *x*-axis represents the average values of the data obtained from both methods. The dashed line in the middle represents the mean difference (mean) between the two conditions, while the solid line in the middle represents the mean difference (d) of 0. The upper and lower solid lines represent the 95% limits of agreement (LoA). The area between the upper and lower lines illustrates the range of confidence interval. It can be observed that the measurement values all fall within the range of the confidence interval, indicating good agreement between the two testing methods. This result confirms the detection accuracy of the constructed homogenous aptamer sensor.

## 3. Materials and Methods

### 3.1. Chemicals and Materials

The AFP aptamer, 5′-ATCAGGTGCAGTTCTCGACTCGGTCTTGATGTGGG-3′, was obtained from Shanghai Sangon Biotech Co., Ltd. (Shanghai, China). The antigen, including preprostate antigen (PSA) and carcinoembryonic antigen (CEA), was obtained from Okai Biotech Co., Ltd. (Beijing, China). Neutrophil gelatinase-associated lipocalin (NGAL) was purchased from Beijing KEY-BIO Biotech Co., Ltd. (Beijing, China). Ethyl orthosilicate (TEOS, 98%), disodium hydrogen phosphate (Na_2_HPO_4_•12H_2_O), and glucose (Glu) were obtained from Aladdin Co., Ltd. (Shanghai, China). Potassium ferrocyanide (K_4_[Fe(CN)_6_]), sodium dihydrogen phosphate (NaH_2_PO_4_•2H_2_O), and potassium ferricyanide (K_3_[Fe(CN)_6_], 99.5%) were obtained from McLean Co., Ltd. (Shanghai, China). Cetyltrimethylammonium bromide (CTAB, 99%), ethanol (99.8%), and sodium hydroxide were obtained from Kermel Reagent Co., Ltd. (Tianjin, China). Ruthenium tribipyridine (Ru(bpy)_3_Cl_2_) and sodium nitrate (NaNO_3_) were provided by Sigma-Aldrich (Shanghai, China). Concentrated HCl (36–38%) was obtained from Shuanglin Chemical Reagent Co., Ltd. (Hangzhou, China). Serum samples were provided by Shanxi Bethune Hospital, Shanxi Academy of Medical Sciences (Taiyuan, China).

### 3.2. Characteriaztions and Instrumentations

The surface and cross-sectional morphology of VMSF was characterized using transmission electron microscopy (TEM) with accelerating voltage of 100 kV (Hitachi HT7700, Hitachi, Ltd., Tokyo, Japan). The cross-sectional morphology of VMSF was investigated using scanning electron microscopy (SEM) at 5 kV (Hitachi SU8010, Japan). To study the elements and functional groups of GO, X-ray photoelectron spectroscopy (XPS) was conducted at 14 kV using a PHI5300 instrument (Physical Electronics, Boston, MN, USA) with Mg Kα source excitation (250 W). Fourier-transform infrared spectroscopy (FTIR) was performed with the KBr plate on a Vertex 70 spectrometer (Bruker, Karlsruhe, Germany). UV-Vis spectra were obtained using a spectrophotometer (UV-2600, Shimatsu, Kyoto, Japan). Zeta potential measurement was performed by using a SZ-100V2 nanoparticle analyzer (HORIBA, Kyoto, Japan) to investigate the charge of the nanomaterials. Autolab (PGSTAT302N) electrochemical workstation (Metrohm, Herisau, Switzerland) was used to perform cyclic voltammetry (CV) and differential pulse voltammetry (DPV) experiments. The testing was performed using a conventional three-electrode system at room temperature. Specifically, Ag/AgCl was used as the reference electrode. Bare ITO or electrodes obtained in the growth of VMSF were employed as the working electrode. Platinum electrode was used as the counter electrode. The parameters set for DPV included pulse amplitude of 0.025 V, pulse time of 0.05 s, step potential of 0.005 V, and interval time of 0.2 s.

### 3.3. Synthesis of VMSF-Functionalized ITO Electrode

VMSF were grown on the surface of the supporting ITO electrode through the electrochemical-assisted self-assembly growth method (EASA) [49]. The precursor solution consisted of ethanol (20 mL), NaNO_3_ (0.1 M, pH = 2.6, 20 mL), TEOS (2.833 g), and CTAB (1.585 g); this was stirred for 2.5 h before use. The clean ITO electrode was put in the precursor solution, and constant current density was applied (−0.7 mA/cm^2^) for 10 s. Subsequently, the nanochannels containing surfactant micelle (SM@VMSF/ITO) were rinsed thoroughly with deionized water and aged at 120 °C for 10 h. To obtain VMSF/ITO electrode with open nanochannels, the obtained SM@VMSF/ITO electrode was stirred with an HCl solution (0.1 M in ethanol) for 5 min to remove SM.

### 3.4. Preparation of Ru(bpy)_3_^2+^/Apt@GO Nanocomposites

Graphene oxide (GO) was synthesized using Hummers’ method [45]. For the preparation of Ru(bpy)_3_^2+^/Apt@GO nanocomposites, Ru(bpy)_3_^2+^ (10 μL, 1 mM), GO (25 μL, 4 mg/mL), and AFP aptamer (10 μL, 100 μM) were incubated at 37 °C for 2 h in PBS (0.01 M, pH = 7.4).

### 3.5. Electrochemical Detection of AFP and Ethical Approval

Different concentrations of the target analyte, AFP, were mixed with the Ru(bpy)_3_^2+^/Apt@GO nanocomposite probes and incubated at 37 °C for 1 h. Then, VMSF/ITO electrode was utilized to measure the oxidation current value of the Ru(bpy)_3_^2+^ probe in the solution via DPV to achieve the quantitative detection of AFP. For real sample analysis, AFP determination in serum samples of four healthy individuals or three liver cancer patients was performed. The study was approved by the ethical committee of Shanxi Bethune Hospital, Shanxi Academy of Medical Sciences (Taiyuan, China, approval number: YXLL-2023-198).

## 4. Conclusions

In summary, a labeling-free and immobilization-free homogenous electrochemical aptamer sensor was constructed for selective and highly sensitive detection of AFP in serum. This was achieved by growing VMSF on a simple, low-cost, and disposable ITO electrode and combining it with a 2D nanorecognition probe. On the one hand, the 2D nanoprobe was easily prepared using this simple procedure, and no complex separation process was needed. On the other hand, the fabrication of a nanochannel-modified electrode was convenient and cost effective. The cost of producing a VMSF/ITO electrode does not exceed USD 1 per square centimeter. Because of the high-density and ultra-small nanochannel, VMSF has size exclusion towards the 2D material probe and significant electrostatic enrichment towards positively charged electrochemical probes. An increase in the concentration of free electrochemical probes in the solution after the combination of AFP with nanoprobes enables sensitive electrochemical detection of AFP. The fabricated homogenous sensor offers a new method for costless, rapid, and sensitive detection of tumor biomarkers in biological samples.

## Figures and Tables

**Table 1:** Comparison between AFP detection performance using different electrodes.

Electrode	Detection Method	Recognition Molecule	Linear Range (ng/mL)	LOD (ng/mL)	Reference
Ab/CP/TiO_2_/FTO	PEC	Antibody	0.1–100	0.03	[43]
P(VT-co-HEMA)-g-GO/Ab_2_/AFP/BSA/Ab_1_/AuNPs-rGO/GCE	EC	Antibody	0.0025–50	1.83 × 10^−4^	[44]
MOF-Ab/AFP/BSA/Apt-SH/Au/PDMS/GCE	EC	Aptamer	0.01–300	0.01	[45]
Ab/EDC-NHS/Fe_3_O_4_/MWCNTs-COOH/AuNPs/GCE	EC	Antibody	10^−3^–10^4^	1.09 × 10^−3^	[46]
AFP/BSA/Ab/rGO@Au@Ru–SiO_2_/GCE	ECL	Antibody	10^−4^–100	3 × 10^−5^	[47]
Poly(DVB-Co-PBA)-AFP/Ab/Ti,Mg@N-CDs/GCE	ECL	Antibody	2.25 × 10^−6^–2250	1.55 × 10^−7^	[48]
VMSF/ITO	EC	Aptamer	10^−3^–1000	0.82 × 10^−4^	This work

CP: conjugated polymers; TiO_2_: titanium dioxide; FTO: fluorine-doped tin oxide conductive glass electrode; P(VT-co-HEMA): poly-(2-hydroxyethyl methacrylate); AuNPs: gold nanoparticles; GCE: glass carbon electrode; MOF: metal–organic framework; Au/PDMS: flexible polydimethylsiloxane/gold; Fe_3_O_4_: ferroferric oxide; MWCNTs-COOH: carboxylated carbon nanotubes; Ru-SiO_2_: Ru(bpy)_3_^2+^-doped silica nanoparticles; rGO@Au: gold nanoparticle-modified reduced graphene oxide; Poly(DVB-Co-PBA): fluorescent microspheres synthesizing based on 4-vinylphenylboronic acid (4-MPBA) and divinylbenzene; Ti,Mg@N-CDs: Ti,N/Mg co-doped carbon dots.

## Data Availability

The data presented in this study are available on request from the corresponding author.

## Sample Availability

Samples of the compounds are available from the authors.
